# Efficient Electrocatalytic Nitrogen Reduction to Ammonia with Electrospun Hierarchical Carbon Nanofiber/TiO_2_@CoS Heterostructures

**DOI:** 10.3390/molecules29246025

**Published:** 2024-12-20

**Authors:** Zhenjun Chang, Fuxing Jia, Xingyu Ji, Qian Li, Jingren Cui, Zhengzheng Liao, Xiaoling Sun

**Affiliations:** 1 College of Materials Science and Engineering, Jiangsu University of Science and Technology, Zhenjiang 212003, China; m18357951288@163.com (F.J.); jixingyu0306@163.com (X.J.); 15070187029@163.com (Q.L.); cuijingren1014@163.com (J.C.); lzzjust0415@163.com (Z.L.); 2 Polytex Engineering Group, Yizheng 225000, China

**Keywords:** electrospinning, carbon nanofibers, CoS, TiO_2_, electrocatalytic properties

## Abstract

As a sustainable alternative technology to the cost- and energy-intensive Haber–Bosch method, electrochemical nitrogen (N_2_) reduction offers direct conversion of N_2_ to NH_3_ under ambient conditions. Direct use of noble metals or non-noble metals as electrocatalytic materials results in unsatisfactory electrocatalytic properties because of their low electrical conductivity and stability. Herein, three-dimensional flexible carbon nanofiber (CNF/TiO_2_@CoS) nanostructures were prepared on the surface of CNF by using electrospinning, a hydrothermal method, and in situ growth. We investigated the behavior of CNFs/TiO_2_@CoS as an electrocatalytic material in 0.1 M sodium sulfate. The highest ammonia yield of the material was 4.61 × 10^−11^ mol s^−1^ cm^−2^ at −0.45 V vs. RHE, and the highest Faraday efficiency, as well as superior long-term durability, was 8.3% at −0.45 V vs. RHE. This study demonstrates the potential of firecracker-shaped nanofiber templates for loading varied noble metals or non-noble metals as a novel development of hybrid composites for electrocatalytic nitrogen reduction.

## 1. Introduction

Ammonia (NH_3_) is an important raw material for urea (a fertilizer) production and the ideal carrier of hydrogen energy [1,2], which is an indispensable part of human production and life. Nowadays, the output of synthetic ammonia is nearly 200 million tons every year in the world. At present, the main process of industrial production of NH_3_ is still the Haber–Bosch (H–B) method. However, its operating conditions are harsh, its energy consumption is large, and its CO_2_ emissions are heavy [3,4,5,6,7]. In recent years, scholars have studied a variety of methods to achieve ammonia synthesis under natural conditions, such as thermal catalytic technology, photoelectric catalytic technology, and biological nitrogenase catalytic technology [8,9,10]. However, there are some deficiencies in the above methods. Therefore, electrocatalytic reduction technology came into being. The electrocatalytic reduction reaction has many advantages, such as being able to be carried out under environmental conditions, being green and environmentally friendly, having low energy consumption, sustainability, and so on. It can also be easier to control the electrocatalytic reduction to obtain the desired products by adjusting the conditions, such as the electrode potential and reaction conditions. An electrocatalytic energy conversion strategy can make the earth’s rich raw materials, such as nitrogen, oxygen, water, and carbon dioxide, into valuable energy, including ammonia, oxygen compounds, and hydrogen. [11,12].

Most of the traditional electrocatalysts are from noble metals, such as Au [13,14,15,16], Pd [17,18,19,20,21,22], Pt [23,24], and Ru [25,26,27,28]. Because noble metals have excellent electrical conductivity and strong binding with reactants, the d-orbital of noble metals can accept lone pair electrons from N_2_ and feed back electrons to the π-orbital of N_2_ molecules, thereby weakening the N≡N bond [29,30]. Therefore, they can be used as efficient electrocatalysts for many reactions. However, due to noble metals’ scarcity and high cost, this technology cannot be developed on a large scale. In recent years, due to the low price of non-noble metals, especially transition metal catalysts, and the rich content of earth, electrocatalytic reduction has become a research hotspot and has great research potential in the field of nitrogen reduction. TiO_2_ is a cheap, non-toxic, and highly electrochemically stable metal oxide material [31]. The growth of its nanorods array structure on the surface of polymer nanofibers can form a very stable firecracker-like structure [32]. It is extremely difficult to destory the carbon nanofibers in an electrochemical environment or at high temperature, which makes it very suitable for being a good conductor skeleton [33]. Studies have shown that TiO_2_ nanosheets can reduce N_2_ to NH_3_ at atmospheric pressure and room temperature, while the Faraday efficiency is only 2.5% lower. In order to improve the NRR performance of TiO_2_, scholars have carried out studies including heteroatom doping and metal atom doping. Cobaltous sulfide (CoS) has become a potential material to replace Pt due to its excellent electrocatalytic performance, high conductivity, abundant raw materials, and low cost. Some works about the composite of CoS-TiO_2_ have been reported. CoS nanosheets can be grown on TiO_2_ powder. It is difficult to control the structure of nanosheets [34]. Furthermore, CoS-TiO_2_ with controllable microstructures immobilized onto CNF nanofibers has seldom been reported. In this paper, this excellent carrier of electrostatic spinning nanofibers was used to solve problems such as the difficulty of the controllable preparation of nanocomposite and hybrid functional materials at micro and nano scales for many years and the difficulty of direct utilization in a macroscopic particle or powder state. Polyacrylonitrile (PAN)-based carbon nanofibers, which are easy to spin electrostatically, were selected as the matrix to carry out our research work, and then the carbon nanohybrid fibers were prepared by using a hydrothermal synthesis method by gradually loading TiO_2_ and CoS on the surface of the nanostructure. The microstructure, composition, and crystallinity of the composite were characterized, and the electrocatalytic nitrogen reduction performance of the hybrid fiber was studied using electrochemical tests.

## 2. Results and Discussion

### 2.1. Morphology and Structure

**Figure 1:**
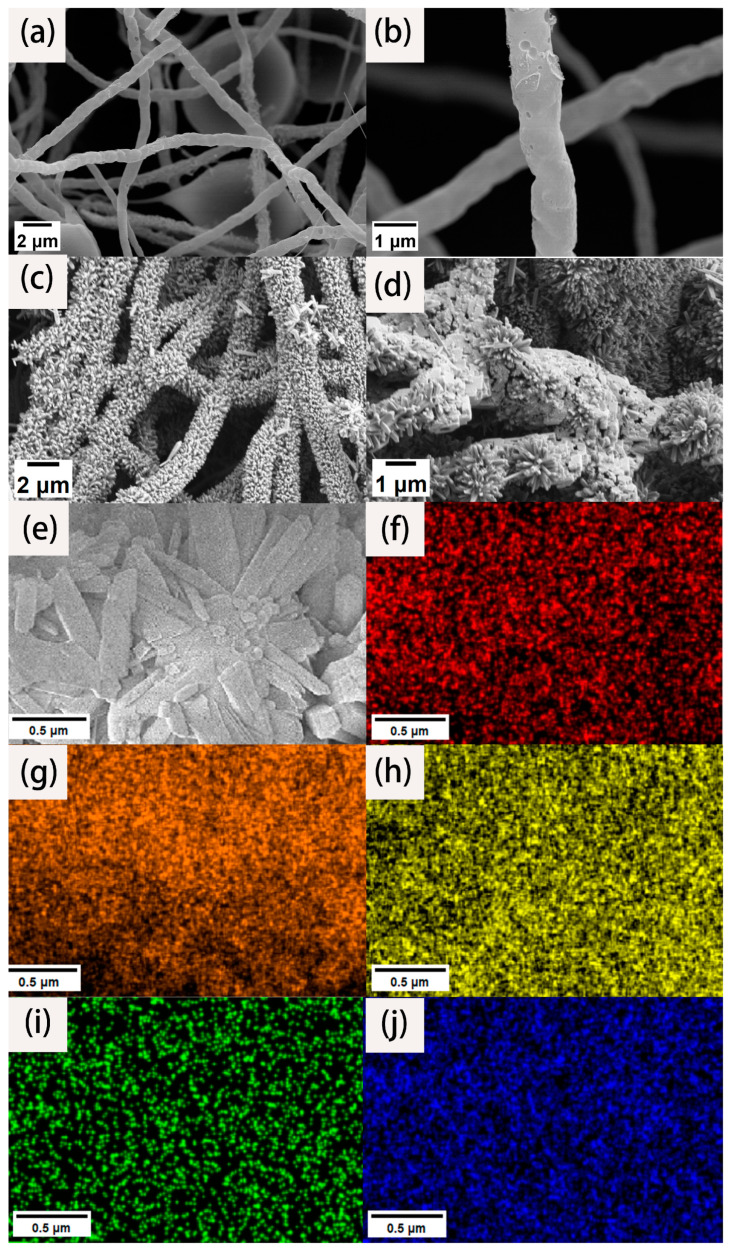
SEM images of CNFs under low (**a**) and high (**b**) magnification. SEM images of CNFs/TiO_2_ (**c**) and CNF/TiO_2_@CoS (**d**). SEM elemental mapping images of CNF/TiO_2_@CoS (**e**), C (**f**), O (**g**), Ti (**h**), Co (**i**), and S (**j**).

To analyze the morphology and microstructure of the materials, high- and low-magnification SEM images were collected. As shown in Figure 1a, we can clearly see that the fiber surface is not smooth, and the length is uniform, with an ultra-high aspect ratio. The fiber diameter is mostly between 600 and 800 nm. There is no entanglement between the fibers. Instead, they are connected to form a three-dimensional network structure. Although these fibers were carbonized at high temperature, they still maintained their fibrous structure. This is due to the fact that the PAN fibers were fully stretched through the action of a high voltage electrostatic field after being sprayed from the electrospinning nozzle. The layers were stacked on the receiving drum to form a fiber membrane. The fiber matrix is not closely integrated. It is a three-dimensional network structure with ultra-high porosity, so there is sufficient space for later load experiments. At the same time, such a network structure is highly accessible for the full infiltration of an electrolyte solution to ensure its effective specific surface area. It can be seen from Figure 1b that there are many holes on the surface of the carbon nanofiber, which is because PTA gasification occurs above 300 °C and escapes with the nitrogen gas flow. The existence of the pore structure improves the specific surface area of the fiber, which increases the active adsorption sites.

Figure 1c shows the SEM images of the CNF/TiO_2_ composites obtained after the hydrothermal reaction. It can be seen that the carbon nanofibers are anchored with an ultrahigh-volume fraction of TiO_2_ nanorods with appearances like firecrackers or bottle brushes. The TiO_2_ nanorods array presents an array distribution on the surface of the CNF almost covering the entire fiber surface. The TiO_2_ nanorods array forms a circular circumferential distribution around the fiber surface. The distribution direction is roughly perpendicular to the fiber surface. The diameter is about 100~200 nm, and the length is about 500~700 nm. Figure 1d shows the SEM images of the synthesized CNF/TiO_2_@CoS three-dimensional composites. It can be seen that the gap and surface of the TiO_2_ nanorods are loaded with CoS nanosheets, which obtained hybrid structures and could enhance the electrocatalytic performance.

EDS was used to quantitatively analyze the element composition and the content of each element on the surface of the composite material. The element mapping results of the material are shown in Figure 1f–j. Figure 1f to f represent the regional distribution spectrum of the C, O, Ti, Co, and S elements on the surface of the composite material. It can be seen from the mapping that the three-dimensional composites are mainly composed of C, O, Ti, Co, and S elements, and the carbon element is mainly distributed at the center of the fiber. According to the SEM results, the TiO_2_ nanorod arrays grow with an ultrahigh-volume fraction, so the corresponding content of the two elements titanium and oxygen is the highest; in the whole scanning field, they have the dense color brightness. Since cobalt sulfide nanosheets grow at the outermost layer of the fiber material, cobalt and sulfur are mostly distributed at the edge of the fiber, which is consistent with the structure of our experimental design.

**Figure 2:**
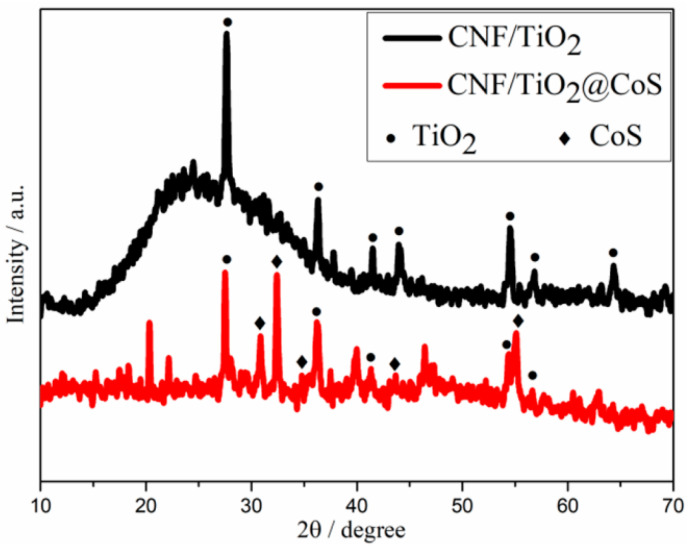
XRD patterns for CNF/TiO_2_ and CNF/TiO_2_@CoS.

XRD measurements were performed to identify the phase and crystal structure of CNF/TiO_2_ and CNF/TiO_2_@CoS (Figure 2). As shown in Figure 2, for the CNF/TiO_2_ sample, the diffraction peaks are located at 2θ of 27.68°, 36.28°, 41.52°, 54.56°, 56.86°, and 69.26°, which correspond to the (110), (101), (111), (211), (220), and (301) diffraction peaks of rutile TiO_2_, respectively (JCPDS: No. 21-1276) [35]. For CNF/TiO_2_@CoS, additional diffraction peaks can be observed at 2θ = 31°, 35°, 47°, and 55°, belonging to the hexagonal CoS (100), (101), (102) and (110) crystal planes, respectively. These results indicate the successful synthesis of CoS nanosheets [36].

**Figure 3:**
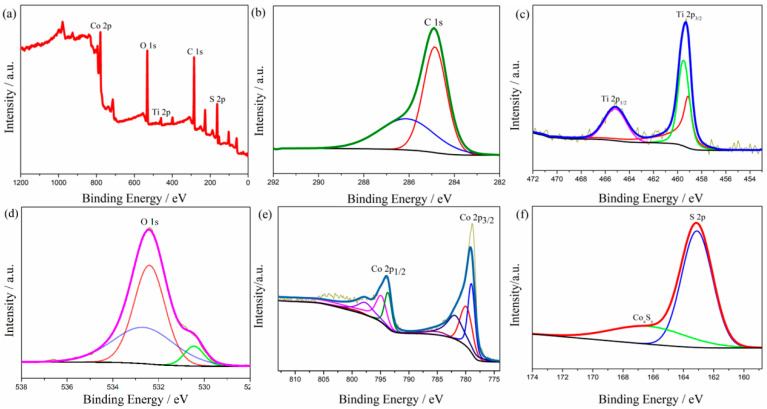
XPS spectra of (**a**) wide-scan, (**b**) C 1s, (**c**) Ti 2p, (**d**) O 1s, (**e**) Co 2p, and (**f**) S 2p for CNF/TiO_2_@CoS.

The chemical composition and valence states were characterized by X-ray photoelectron spectroscopy (XPS), and the corresponding results are shown in Figure 3. The survey spectrum displays the existence of C, Ti, O, Co, and S elements in the composite (Figure 3a). As shown in Figure 3b, the bond energy of the main peak in the detail in the figure of C 1s is 284.60 eV [37], which corresponds to the carbon–carbon single bond or carbon–carbon double bond contained in the skeleton structure of the fiber after carbonization. In addition, the characteristic peak of the carbon–oxygen double bond appeared at a 286.20 eV binding energy, which may be because of the existence of hydroxyl residues in the composite after the hydrothermal reaction. Figure 3c shows that the binding energy of Ti 2p is 459.4 eV and 465.2 eV, corresponding to Ti 2p_3/2_ and Ti 2p_1/2_, respectively [38]. The difference in binding energy is 5.8 eV, indicating the existence of tetravalent titanium ions, which proves the existence of titanium dioxide. Figure 3d shows the detail of the spectrum of O 1s. The characteristic peak at 530.5 eV is the Ti–O bond of TiO_2_ [39]. Figure 3e is the detailed spectrum of Co 2p. The characteristic peak at 778.8 eV is a Co–S bond, which proves that cobalt and sulfur form cobalt sulfide [40]. The peak at 163.1 eV is the typical characteristic of S 2p of CoS. The broad peak at 165~170 eV may come from a small amount of nonstoichiometric Co_x_S_y_ [36].

### 2.2. Electrochemical Measurement

**Figure 4:**
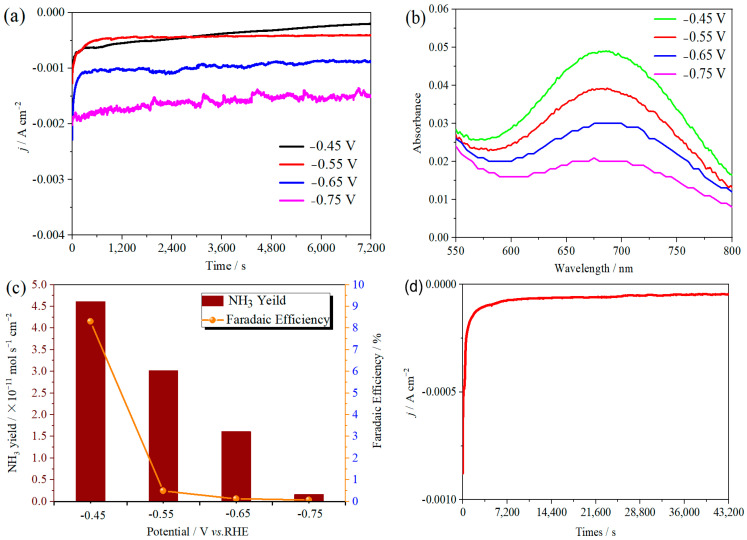
(**a**) Time-dependent current density curves, (**b**) UV–vis spectra, and (**c**) ammonia yields and Faradaic efficiencies of the CNF/TiO_2_@CoS nanofibrous membrane at different potentials. (**d**) Time-dependent current density curve for CNF/TiO_2_@CoS in 0.1 M Na_2_SO_4_ electrolyte at −0.45 V vs. RHE for 12 h.

**Figure 5:**
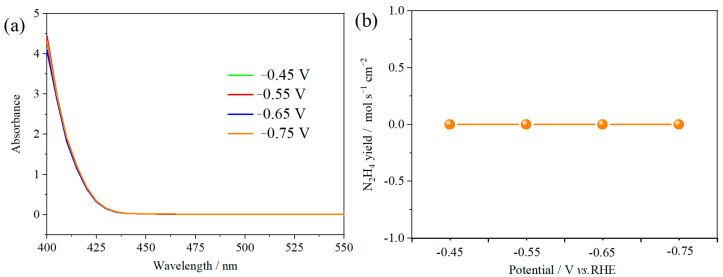
(**a**) Absorbance curves (**b**) N_2_H_4_ yield of p-dimethylaminobenzaldehyde indicator after electrolysis for 2 h at different potentials.

**Figure 6:**
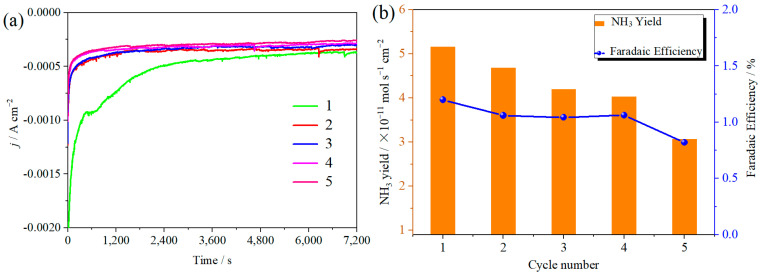
(**a**) Current density time curves; (**b**) ammonia yield and Faraday efficiency of CNF/TiO_2_@CoS in 0.1 M Na_2_SO_4_ at −0.45 V vs. RHE. for 2 h and five times.

**Figure 7:**
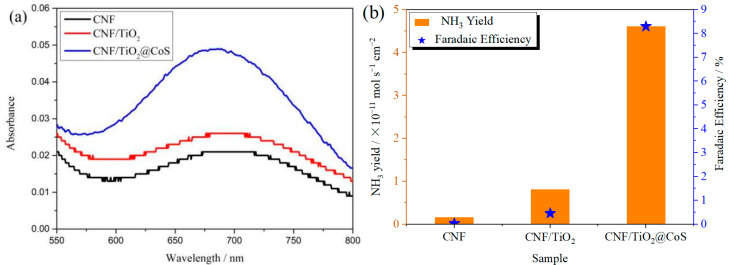
(**a**)Absorbance curves and (**b**)NH_3_ yields and FEs of CNF, CNF/TiO_2_, and CNF/TiO_2_@CoS nanofiber membranes after electrolysis at −0.45 V vs. RHE for 2 h.

**Figure 8:**
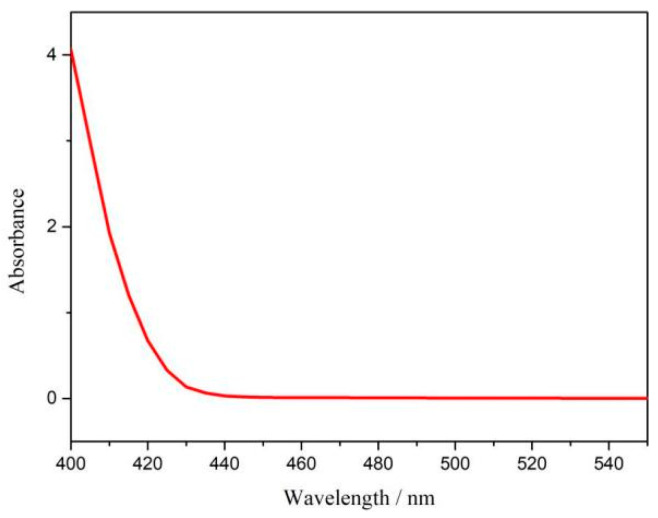
Absorbance curve of p-dimethylaminobenzaldehyde indicator at optimum potential after electrolysis for 12 h.

The ambient nitrogen reduction was conducted in a H-type electrolytic cell, in which a CNF/TiO_2_@CoS nanofibrous membrane (0.5 × 0.5 cm^2^) was directly used as the working electrode. The electrolyte (0.1 M Na_2_SO_4_) was saturated with high-purity (99.999%) nitrogen for the polarization test. The potentiostatic polarization curves are shown in Figure 4a. After 2 h electrolysis, the electrolyte in the cathodic compartment was sampled and subjected to the indophenol blue method to determine the NH_3_ yield. After being stained with the indophenol indicator, it was found that the concentration of the NH_3_ solution was linearly related to its absorbance. Furthermore, the UV–vis spectra of the electrolytes obtained at different electrolytic conditions from −0.45 to −0.75 V vs. RHE are shown in Figure 4b. It was found that the highest NH_3_ yield was 4.61 × 10^−11^ mol s^−1^ cm^−2^ at −0.45 V vs. RHE. At the same time, the highest Faradaic efficiency was 8.3% at −0.45 V vs. RHE, as shown in Figure 4c. As the association mechanism of the electrocatalytic nitrogen reduction mechanism can be divided into the two ways of distal hydrogenation and alternate hydrogenation, according to the different hydrogenation sequence, hydrazine may be produced as a by-product [41]. Thus, to test the selectivity of the CNF/TiO_2_@CoS nanomaterials, the electrolyte after 2 h electrolysis was sampled and subjected to the Watt and Chrisp method to determine the presence of N_2_H_4_ [36]. As shown in Figure 5, hydrazine was not detected from the electrolyte, and the N_2_H_4_ yield in the electrolyte was nearly zero, which indicated the exclusive selectivity of the CNF/TiO_2_@CoS nanofibrous membrane toward NH_3_ [36]. The durability and repeatability over 12 h at the optimal potential were investigated. As shown in Figure 4d, during the long-term test over 12 h, the current density did not decay much, which is attributed to the good structural stability of the composites. After waiting for 20 min under dark conditions, the electrolytes were subjected to a UV test, and the absorbance curves are shown in Figure 5. There was no indication of absorbance at 458 nm, suggesting that the yield of hydrazine in electrolyte is almost zero and that the catalyst has good selectivity towards ammonia. As shown in Figure 6, after five repeated test cycles at the optimum potential of −0.45 V vs. RHE, the ammonia yield and Faraday efficiency of the nanofiber membrane slightly decreased, indicating that the electrocatalyst has good reusability. To compare the electrocatalytic performance of different working electrodes of CNF, CNF/TiO_2_, and CNF/TiO_2_@CoS, the UV–vis spectra of the electrolytes collected at −0.45 V vs. RHE are presented in Figure 7. It can be seen that the highest NH_3_ yield and the higher FE were observed with the presence of CoS. Figure 8 shows the absorbance curve of hydrazine after the long-term test of the composite material. It can be seen from the figure that no by-products were produced in the electrocatalytic process, which further indicates that the electrocatalyst has a good ammonia selectivity.

### 2.3. Mechanism Analysis of the Electrocatalytic Reduction of Nitrogen

**Figure 9:**
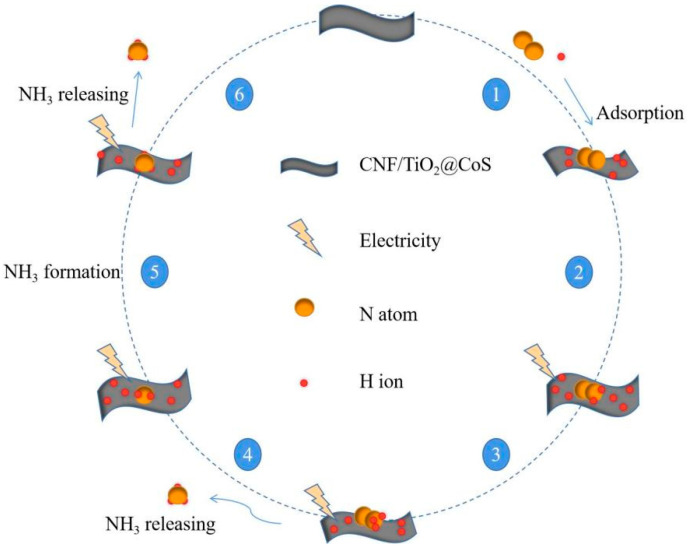
Proposed pathway for the NH_3_ synthesis using CNF/TiO_2_@CoS catalyst.

Since hydrazine was not found, we can speculate that this experiment’s result was based on the remote hydrogenation pathway to achieve electrocatalytic synthesis of ammonia [42], and a possible mechanism diagram of its reaction is shown in Figure 9.

In the remote hydrogenation pathway [43], the N atom at the remote end first hydrogenates until the NH_3_ at the far end is released, and the remaining nitrogen atoms located on the surface of the catalyst hydrogenate again, releasing a second NH_3_ molecule. This is a multi-step process of proton-coupled electron transfer involving a mechanistic reaction, as shown in Equations (1)–(8) below, wherein ‘*’ represents the catalyst.

* + N_2_
→ *N_2_ (1)

*N_2_ + 6(H^+^ + e^−^) → *NNH + 5(H^+^ + e^−^) (2)

*NNH + 5(H^+^ + e^−^) → *NNH_2_ + 4(H^+^ + e^−^) (3)

*NNH_2_ + 4(H^+^ + e^−^) → *N + NH_3_ + 3(H^+^ + e^−^) (4)

*N + 3(H^+^ + e^−^) → *NH+ 2(H^+^ + e^−^) (5)

*NH + 2(H^+^ + e^−^) → *NH_2_ + (H^+^ + e^−^) (6)

*NH_2_ + (H^+^ + e^−^) → *NH_3_ (7)

*NH_3_
→ NH_3_ + * (8)

## 3. Experimental Section

### 3.1. Materials

Polyacrylonitrile (PAN, Mw = 80,000), terephthalic acid (PTA), N,N-dimethylformamide (DMF), titanium powder, cobalt chloride hexahydrate (CoCl_2_·6H_2_O), thiourea, ammonium chloride (NH_4_Cl), sodium hypochlorite (NaClO), sodium sulfate (Na_2_SO_4_), hydrazine hydrate (N_2_H_4_·H_2_O), sodium salicylate (C_7_H_5_O_3_Na), sodium hydroxide (NaOH), and ethanol (CH_3_CH_2_OH), were received from Beijing Chemical Corporation (Beijing, China). Sodium nitroferricyanide (III) dihydrate(Na_2_Fe(CN)_5_NO·2H_2_O), para-(dimethylamino) benzaldehyde (C_9_H_11_NO), and concentrated hydrochloric acid were obtained from Sinopharm Chemical Reagant Co., Ltd., (Shanghai, China).

### 3.2. Preparation of Porous Carbon Nanofiber (CNF) Membrane

Firstly, the spinning precursor solution with 9 wt% PAN and 4 wt% PTA in DMF was prepared [44]. Then, the prepared precursor solution was injected into a 1 mL syringe using a micro-injection pump. The propulsion speed of the injection pump was adjusted to 1 mL/h, and the power supply voltage was set to 10.5 kV. The distance between the drum receiver and syringe needle was about 15 cm, so that the drum maintained a uniform rotation, and then the electrospinning was started. After several hours of electrospinning, a white PAN nanofiber membrane was evenly coated on the roller. The PAN nanofiber membrane was removed and cut into appropriate sizes. The membrane was placed into a quartz boat and placed in a high-temperature tubular furnace. The pre-oxidation process was carried out in air to 260 °C and kept for 2 h (heating rate 5 °C/min), and then high-purity nitrogen was slowly introduced. With the same heating rate, the temperature was increased to 800 °C and kept for about 2 h for the carbonization. The tubular furnace was slowly cooled to room temperature, and the nitrogen was stopped afterwards. The sample was taken out. It can be seen that the CNF membrane sample changed from white to black, and its macromorphology was still maintained as a membrane.

### 3.3. Preparation of CNF/TiO_2_

TiO_2_ nanorod arrays were grown on the surface of CNFs. The specific operation was as follows: Firstly, 45 mg titanium powder was weighed and put into a high-pressure hydrothermal reactor, and then 3 mL concentrated hydrochloric acid (37%) and 33 mL deionized water were added into the container and stirred with a glass rod. Then, the as-prepared CNF was put into the hydrothermal reactor and was completely immersed in the above-mentioned solution. The reactor was sealed, heated to 160 °C, and kept for 16 h. Afterwards, it was slowly cooled to room temperature. The sample was taken out, carefully washed several times with deionized water, and finally dried at 60 °C. It can be clearly seen that a layer of silver-gray material was loaded on the surface of the black CNF, indicating that TiO_2_ nanorod arrays were successfully ‘planted’ on the CNF surface. Thus, CNF/TiO_2_ composites were obtained.

### 3.4. Preparation of CNF/TiO_2_@CoS

The growth of CoS nanosheets was prepared by hydrothermal synthesis. The experimental synthesis steps were as follows: 4 g CoCl_2_·6H_2_O was dissolved in 30 mL deionized water, then 1.3 g thiourea was added and mechanically stirred for 20–30 min to obtain a pink clarification solution; the solution was transferred to a 50 mL high-pressure reactor, and then the CNF/TiO_2_ material obtained from the last step was completely immersed in the solution. The high-pressure reactor was sealed and placed in a constant-temperature oven. After heating to 170 °C for 10 h, the constant-temperature reaction was slowly cooled to room temperature. The sample material was carefully taken out and rinsed repeatedly with deionized water and ethanol several times. The obtained sample was thoroughly dried in an oven at 80 °C. In the end, the color of the fiber membrane surface became darker. The CNF/TiO_2_@CoS three-dimensional composite material was obtained.

### 3.5. Material Characterization

An emission scanning electron microscope (FESEM, Hitachi S-4800 and FEI QUANTA 250) was used for the analysis of the morphology of the samples. Energy-dispersive X-ray spectroscopy (EDS) was used for the analysis of the composition of the materials. X-ray diffraction (XRD, Bruker D8 Advance) with Cu Kα irradiation (λ = 1.5406 Å) was used for examining the crystalline nature of the nanofiber samples. The surface property of the prepared samples was determined by X-ray photoelectron spectroscopy (XPS, ESCALAB250Xi) on a Kratos AXIS HIS spectrometer (Al Kα X-ray source (1486.6 eV)). UV–vis spectra were acquired on a Purkinje TU-1950 ultraviolet-visible (UV–vis) spectrophotometer. The humidity of the laboratory was about 60%.

### 3.6. Electrochemical Measurements

N_2_ reduction experiments of the samples were carried out at atmospheric pressure and room temperature in an H-type double-chamber electrolytic cell, which was separated by a Nafion 212 membrane. Firstly, the membrane was protonated by first boiling it in deionized water for 1 h and treating it in an H_2_O_2_ (5 wt%) aqueous solution at 80 °C for another 1 h. Secondly, the membrane was soaked in H_2_SO_4_ (0.5 M) for 3 h at 80 °C and finally in deionized water for 6 h. Furthermore, the electrochemical experiments were carried out with an electrochemical workstation (CS350H electrochemical workstation) using a three-electrode configuration with a saturated calomel electrode (SCE) (with saturated KCl electrolyte, platinum plate electrode, and prepared electrodes as the reference electrode, counter electrode, and working electrode, respectively. The potentials reported in this work were converted to the RHE scale via calibration with the following equation: E_RHE_ = E_Hg_/_HgO_ + 0.059 × pH + 0.245 V. The presented current density was normalized to the geometric surface area. In the electrochemical N_2_ reduction, the chronoamperometry tests were conducted in an N2-saturated Na_2_SO_4_ (0.1 M) solution.

### 3.7. Determination of NH_3_

The produced NH_3_ was spectrophotometrically confirmed by the indophenol blue method. Specifically, 4 mL electrolyte was obtained from the cathodic chamber and mixed with NaOH (0.75 M), an oxidizing solution (50 µL) containing NaClO (ρCl = 4~4.9), a 500 µL coloring solution containing 0.4 M C_7_H_6_O_3_Na and 0.32 M NaOH, and a 50 µL catalyst solution (1 wt% Na_2_[Fe(CN)_5_NO]·2H_2_O) for 2 h. The concentration–absorbance curve was calibrated by using a standard NH_3_ solution with a series of concentrations. The corresponding calibration curves are shown in Figure S1. The fitting curve (Y = 0.81X + 0.02, R^2^ = 0.995) showed a good linear relation between absorbance values and NH_3_ concentration.

### 3.8. Determination of Faraday Efficiency (FE) and NH_3_ Yield

The electrocatalytic performance was evaluated by measuring the ammonia production rate and Faraday efficiency (FE) of the composite as the nitrogen reduction electrocatalyst.

The NH_3_ yield was calculated using the following equation:

NH_3_ yield = (C_NH3_ × V)/(17 × t × A)

where C_NH3_ is the ammonia concentration (μg mL^−1^), V is the electrolyte volume (40 mL), t is the electrolysis time (2 h), and A is the working electrode area (0.5 × 0.5 cm^2^).

The FE was obtained by dividing the ammonia produced during the experiment by the total charge applied to the electrode (Q). Since the generation of an ammonia molecule requires three electrons (N_2_ + 6H^+^ + 6e^−^ → 2NH_3_), the FE could be calculated as follows:

FE = (3F × C_NH3_ × V)/(17 × Q)

where F is the Faraday constant (96,485 C mol^−1^).

### 3.9. Determination of N_2_H_4_

Hydrazine may be produced as a by-product of electrochemical N_2_ reduction. In this experiment, the Watt and Chrisp methods were used to measure its existence. An N_2_H_4_ solution with concentrations of 0.0, 0.2, and 0.4 µg mL^−1^ was prepared as the standard solution; 4 mL of standard solution with different concentrations and 4 mL of chromogenic agent were added. The solution was kept at room temperature for 20 min before applying UV-visible light. Figure S2 shows the UV absorbance curve of the standard concentration hydrazine solution. The maximum absorption wavelength was 458 nm [45].

## 4. Conclusions

In conclusion, 3D CNF/TiO_2_@CoS nanofibers were successfully prepared by combining electrospinning, a hydrothermal process, and in situ growth. The 3D CNF/TiO_2_@CoS composites were characterized by large specific surface areas and well-dispersed active sites. The XRD analysis showed that the TiO_2_ prepared by this method was the rutile type. The electrocatalytic performance of the fiber membrane was explored, and the ammonia yield was compared for the same time and at different voltages. The highest ammonia yield was 4.61 × 10^−11^ mol s^−1^ cm^−2^ at −0.45 V vs. RHE, and the highest FE was 8.3% at −0.45 V vs. RHE. The results of the long-term stability test and the reproducibility test showed that the composite has excellent catalytic stability and excellent ammonia selectivity without a hydrazine by-product, which provides a new idea for the electrocatalytic reduction of ammonia under ambient conditions. We will study the photo-electrocatalytic applications of the materials. In the future, we will use other non-noble metals to grow on CNF/TiO_2_ for electrocatalytic applications. With the selection of an appropriate skeleton and immobilization of all kinds of tailor-made non-noble metals, the design can provide a powerful tool for various electrocatalysts, including NRR, CO_2_RR, OER, ORR, HER.

## Data Availability

The data presented in this study are available on request from the corresponding author.

## Supplementary Materials

The following supporting information can be downloaded at: www.mdpi.com/article/10.3390/molecules29246025/s1, Figure S1. (a) UV-vis spectra and (b) calibration curve of NH3 solutions with different concentrations. Figure S2. UV absorption curves after coloration of paradimethylaminobenzaldehyde indicator with hydrazine standard solution.
