# Supplementary material for: Efficient Electrocatalytic Nitrogen Reduction to Ammonia with Electrospun Hierarchical Carbon Nanofiber/TiO2@CoS Heterostructures
Source: Molecules. 2024 Dec 20;29(24):6025. doi: 10.3390/molecules29246025 (PMC11677930; doi:10.3390/molecules29246025)
Supplement: Supplementary file 1 [file molecules-29-06025-s001.zip › molecules-3326392-supplementary.pdf]

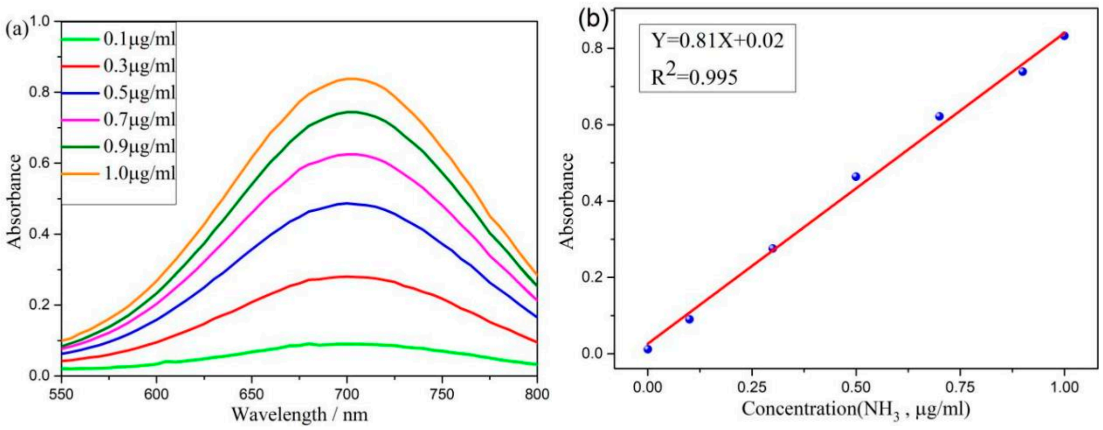

Figure S1. (a)UV-vis spectra and(b)calibration curve of NH_3 solutions with different concentrations.

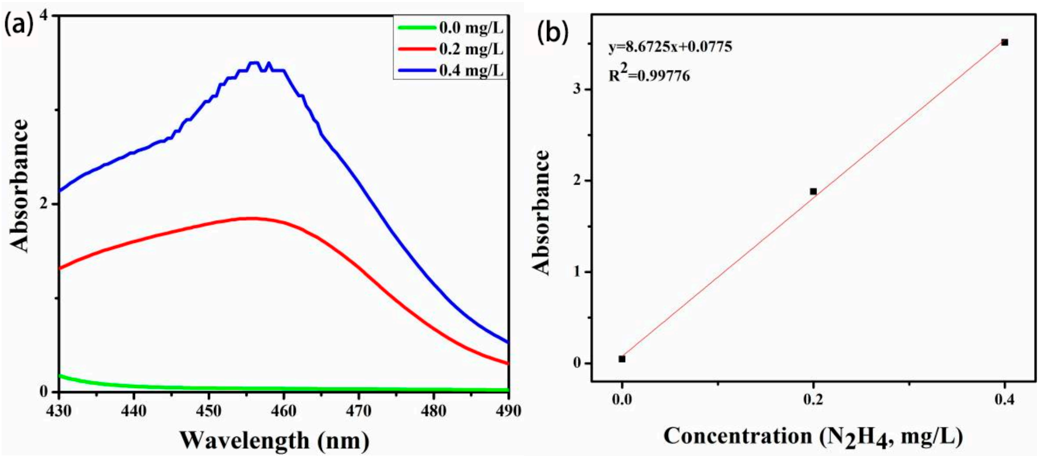

Figure S2. UV absorption curves after coloration of paradimethylaminobenzaldehyde indicator with hydrazine standard solution.
